# Predictors of HIV testing among women experiencing intimate partner violence in the Central Region of Ghana

**DOI:** 10.1371/journal.pgph.0000376

**Published:** 2022-05-04

**Authors:** Beatrice Adwoa Afari, Juliana Yartey Enos, Deda Ogum Alangea, Adolphina Addo-Lartey, Adom Manu

**Affiliations:** 1 Department of Population Family and Reproductive Health, School of Public Health, College of Health Sciences, University of Ghana, Legon, Accra, Ghana; 2 Department of Epidemiology, Noguchi Memorial Institute for Medical Research, College of Health Sciences, University of Ghana, Legon, Accra, Ghana; 3 Department of Epidemiology and Disease Control, School of Public Health, College of Health Sciences, University of Ghana, Legon, Accra, Ghana; University of the Punjab, PAKISTAN

## Abstract

HIV testing, which is important for the control of the HIV pandemic, has been hampered by several factors including Intimate Partner Violence (IPV), resulting in low uptake. This study sought to determine the predictors of HIV testing among women experiencing IPV. Secondary analysis of data generated from a cross-sectional mixed-method unmatched cluster-randomized controlled trial designed to evaluate a multi-faceted community intervention to reduce the incidence of IPV in Ghana was done (N = 2000). Logistic regressions were performed to determine the predictors of HIV testing among women experiencing IPV, using the trial baseline data. The prevalence of HIV testing among women exposed to IPV in the study setting was 42.4%. Less than a third of the respondents (30.2%) had ever used condom and 96.6% had unemployed partners. Age, educational attainment, employment, residence and condom use were found to be significant predictors of HIV testing among women experiencing IPV. Women aged 25–39 years were more than twice as likely to test for HIV (AOR:2.41; 95%CI:1.45–4.02) than those above 45 years. Women with formal education (Junior-High—AOR:2.10; 95%CI:1.42–3.12; Senior-High—AOR:3.87; 95%CI:2.07–7.26); who had ever used condom (AOR:1.42; 95%CI:1.05–1.93); those reporting life satisfaction (AOR:1.44; 95%CI:1.08–1.92); and coastal residents (AOR:1.97; 95%CI:1.45–2.67) were more likely to test for HIV than those who did not and inland residents. However, employed women (AOR:0.66; 95%CI:0.45–0.96) were less likely to test for HIV than unemployed women. Less than half of the women exposed to IPV had tested for HIV. Socioeconomic disadvantages related to age, education, employment, residence and life satisfaction predicted HIV testing among women exposed to IPV. Considering the vulnerability of women experiencing IPV to HIV infection, strategies to improve uptake of HIV testing must tackle contextual socioeconomic factors that hinder access to services.

## Introduction

Human Immunodeficiency Virus (HIV) infection continues to be of global public health importance with a significant burden in sub-Saharan Africa [1]. More than half of the over 37 million people infected with HIV are women in their reproductive ages [1]. HIV testing and screening services are crucial to the control of infections and form a central component of holistic control efforts aimed at reducing the prevalence of HIV and related deaths, globally [2]. Efforts at increasing testing rates among the general population and vulnerable groups such as women experiencing intimate partner violence (IPV) are essential to meeting the goals of the HIV epidemic control [2].

In general, about 20% of persons infected with HIV remain unaware of their HIV status with disproportionate figures from low- and middle-income countries [2]. Inadequate testing tends to mask the burden of HIV and contributes to the continuous spread of the virus, especially among vulnerable groups, including women experiencing IPV [3]. In Ghana, for instance, the low uptake of testing services made it difficult for the country to achieve the global target of diagnosing at least 90% of people with HIV infection by the end of 2020 [4]. Among the populations at risk of HIV and other sexually transmitted infections, women who experience intimate partner violence are at high risk. A WHO (2013) study on the health burden associated with violence against women, revealed that women who have been abused either physically or sexually were 1.5 times at risk of sexually transmitted infection of HIV [5].

Intimate partner violence (IPV), which includes physical, sexual and emotional abuse as well as other controlling behaviours by an intimate partner, occurs globally [6]. According to WHO (2021), 1 in 3 women globally experienced some form of IPV in their lifetime [6]. IPV rates ranging from 26.5% to 48% with an average of 25.7% have been reported for sub-Saharan African countries including Egypt, Kenya, Malawi, Rwanda and Zambia [7]. IPV prevalence rate of 59.7% among women with and without HIV in the Southern part of Ethiopia [8] has also been reported. These estimates are similar to those found among HIV positive women in Uganda ranging from 44% to 56% [9]. In Ghana, findings from the Demographic and Health Survey (2008) indicated an IPV prevalence rate of 38.7% among ever-married women between the ages of 15 and 49 years across the country [10], and the Ghana Family Life and Health Survey (2015) reported an IPV prevalence of 22.7% in the 12 months prior to the survey [11].

IPV is among the structural barriers to achieving HIV-related goals such as testing, treatment and adherence to treatment [12]. Women who experience IPV, in addition to having increased risks of contracting the virus, may have challenges in accessing HIV testing and treatment services due to gaps resulting from gender inequalities such as economic vulnerability, male-centered household decision-making and potential consequences of accessing related services and stigma [12]. The fear or perpetration of violence by intimate partners act as barriers to utilizing testing services or disclosing their test results, further lowering testing rates among women exposed to IPV [12]. HIV testing among this population is thus critical for early detection and prevention efforts, which also serves to ensure they do not propagate the infection to others.

There have been interventions to both improve HIV testing and address the incidence of IPV in sub-Sahara Africa, as the determinants of HIV and IPV are closely interrelated [13, 14]. A comprehensive understanding of the factors associated with HIV testing among women who experience IPV is essential to guide programming and inform targeted interventions to address the compounding effect of HIV and IPV among women, and the barriers to accessing services. This study, therefore, sought to determine the predictors of HIV testing among women experiencing IPV in the Central region of Ghana.

## Methods

### Ethics statement

This study is based on the analysis of secondary data from an original study (RSS trial protocol registered on ClinicalTrials.gov (Identifier: NCT03237585)), that obtained ethical approval from the Institutional Review Board of the Noguchi Memorial Institute for Medical Research of the University of Ghana (Protocol ID # 006/15-16) and the South African Medical Research Council’s Ethics Committee (Protocol ID # EC031-9/2015) [15]. Hence, the secondary data analyses did not require additional ethical approval from an Ethical Review Board. However, permission was obtained for the use of data for this study from the original study investigators, who are co-authors on this manuscript. The original study obtained written informed consent from all participants. Prior to participation in the survey, researchers discussed the study’s consent form with respondents, which included explanation of study objectives, potential risks, benefits, confidentiality of information and voluntariness of participation. Respondents were assured of anonymity with the use of unique identification codes that could not be traced to them. Thus, the data used for this study was fully anonymized before being accessed for this study and identity of the respondents is not known to the authors of this study. The secondary data obtained was strictly used to address the objectives of this study for which it was intended and nothing else.

### Study design and population

A cross-sectional study using secondary data from a mixed-method unmatched cluster-randomized controlled trial carried out in four districts (two inland and two coastal) in the Central Region of Ghana was conducted. The four districts were the Upper Denkyira, Agona, Abura-Asebu-Kwamankese and Komenda-Edina-Egyafo-Abirem municipal districts. Upper Denkyira and Agona districts were considered “inland” districts, whilst Abura-Asebu-Kwamankese and Komenda-Edina-Eguafo-Abirem (KEEA) municipal were considered as “coastal” areas of the region. The trial assessed the Rural Response System (RRS), which used Community-Based Action Teams ‘COMBAT’ in preventing violence against women (VAW) in Ghana. The study involved 2000 randomly selected women aged 18–49 years living in 40 localities within the four districts selected for the study. Although the prevalence of HIV testing was assessed for the whole ‘COMBAT’ study population of 2000 women at baseline, only data for 1008 of the women in the study, who experienced any form of IPV were used for this study on the predictors of HIV testing among IPV-exposed women. Respondents were interviewed in English and Twi/Fanti, the local dialects of the study area.

### Study variables

#### Dependent variables

The primary outcome variable for this study was ‘HIV testing’ among women who experienced ‘any form of IPV’, which includes physical, sexual, emotional and economic IPV. HIV testing among women who experienced any form of IPV was defined as a binary outcome variable and categorized as ‘0’ for those who had never tested for HIV and ‘1’ for those who had ever tested for HIV.

#### Independent variables

The independent variables considered in this study were socio-demographic and sexual-behavior factors. The socio-demographic characteristics of respondents included age, residence, number of years respondent had lived in the community, highest level of education, educational level of respondents’ partners, movement of respondent or travel outside the community for work, employment status in the 12 months preceding the survey, employment status of partner and reported satisfaction with life. The sexual-behavior characteristics considered were the respondent’s age at first sex, first sexual partner, whether the respondent had ever used a condom, number of main sexual partners, number of other sexual partners, and the number of transactional sex the woman had ever engaged in. Other data collected for the original study, which were not considered salient for this study and not used include mental health and substance use; household food insecurity; gender norms/attitudes; and childhood trauma experience. The decision on which variables to include in the study were guided by the literature. For example, mental health and substance abuse have been shown to have a strong relationship with IPV, and gender norms and attitudes have also been shown to influence IPV. However, these variables were not of interest in the current study.

### Data analysis

Descriptive statistics were used to summarize the characteristics of women in the study, including whether they had ever tested for HIV or not. Pearson’s chi-square tests were done to determine associations of the socio-demographic and sexual behaviour characteristics with “HIV testing” as an outcome variable. Logistic regression was then used to estimate adjusted odds ratios and 95% confidence intervals for variables associated with HIV testing among women experiencing IPV. The Least Absolute Shrinkage and Selection Operator (LASSO) with a 10-fold cross validation method was used to select the most predictive variables to include in the multivariable logistic regression model. The LASSO approach selects the minimum number of variables that best predicts the outcome and, in the process, avoids over-selection and under-selection of variables. The crude and adjusted odds ratios and respective confidence intervals and p-values of the variables used in the final model are presented. The goodness of fit test of the final model had a non-significant p-value of 0.4093 indicating an appropriately fitted model. The mean variance inflation factor (VIF) was 2.76 (range: 1.32 to 7.08), which is less than 10 indicating an acceptable level of multicollinearity among the variables used in the multivariable logistic regression model. All statistical analyses were performed using STATA IC Lasso Reference Manual version 16.

## Results

### Characteristics of study participants

The mean age of the respondents for this study was 32.1 ± 8.59 years. About 55.5% of the respondents lived in inland areas and 44.5% in coastal areas. The study population was quite stable with 18.9% having lived in their communities for more than 29 years. The mean number of years that respondents had lived in their community was 17.4 ±12.36 years. Most of the respondents (64%) had more educated male partners. However, 14.6% of the respondents were more educated than their male partners. Also, 83.0% were employed in the past 12 months and 96.6% had partners who were unemployed. About 66.7% of respondents reported not being satisfied with life. Regarding the sexual-behaviour characteristics, a few respondents (11.1%) had their initial sexual experience with their husbands, while most respondents (77.1%) had their first sexual experience with their boyfriends. Most respondents (87.3%) had one sex partner and 17.9% had been involved in two or more transactional sexual relations. Only 30% of respondents had ever used condom. Regarding the prevalence of HIV testing, the study found that less than half (43.3%) of all study participants involved in the baseline assessment for the cRCT had ever tested for HIV in their lifetime. Prevalence of HIV testing among respondents who had experienced IPV was 42.4% compared to 44.2% among those who had not experienced any form of IPV. The sociodemographic and sexual-behaviour characteristics of study participants are presented in Table 1.

**Table 1:** Characteristics of study participants (N = 1008).

Characteristics of respondents	N (%)
**Socio-demographic characteristics**	
**Age (years)**	
<25	220 (21.83)
25–29	221 (21.92)
30–34	182 (18.06)
35–39	136 (13.49)
40–44	128 (12.70)
45–49	121 (12.00)
Mean age ± SD	32.09 ± 8.59
**Area of residence**	
Coastal	449 (44.54)
Inland	559 (55.46)
**Years lived in community**	
<10	329 (32.64)
10–19	221 (21.92)
20–29	267 (26.49)
>29	191 (18.95)
Mean years lived ± SD	17.39 ± 12.36
**Highest level of education**	
None	227 (22.52)
Primary	254 (25.20)
Junior high	451 (44.74)
Senior high	66 (6.55)
Post senior high	10 (0.99)
**Current partner’s education level**	
Same	216 (21.43)
Woman more educated	147 (14.58)
Male partner more educated	645 (63.99)
**Ever moved or travelled from usual residence for work**	
No	545 (54.07)
Yes	463 (45.93)
**Employed in the past 12 months**	
No	171 (16.96)
Yes	837 (83.04)
**Partner employed**	
No	974 (96.63)
Yes	34 (3.37)
**Self-reported life satisfaction**	
Not satisfied	672 (66.67)
Satisfied	336 (33.33)
**Sexual-behaviour characteristics**	
**Age at first sex (years)**	
<16	146 (14.48)
16–19	590 (58.53)
20 years and above/Never had sex	272 (26.98)
**First sex partner**	
Husband	112 (11.11)
Boyfriend	777 (77.08)
Others/Never had sex	119 (11.81)
**Ever used condom**	
No	704 (69.84)
Yes	304 (30.16)
**No. of main sex partners in last 12 months**	
0	108 (10.71)
1	880 (87.30)
>1	20 (1.98)
**No. of other sex partners in last 12 months**	
0	927 (91.96)
1	64 (6.35)
>1	17 (1.69)
**No. of main sex partners in lifetime**	
0	10 (0.99)
1	317 (31.45)
>1	681 (67.56)
**No. of other sex partners in lifetime**	
0	813 (80.65)
1	127 (12.60)
>1	68 (6.75)
**Number of transactional sex engaged in**	
0	753 (74.70)
1	75 (7.44)
>1	180 (17.86)
**Type of IPV experienced**	
Economic IPV	202 (20.04)
Emotional IPV	734 (72.82)
Physical IPV	643 (63.79)
Sexual IPV	370 (36.71)
**HIV testing in lifetime**	
HIV testing among women exposed to IPV (N = 1008)	
No	581 (57.64)
Yes	427 (42.36)
HIV testing among women not exposed to IPV (N = 992)	
No	554 (55.85)
Yes	438 (44.15)
HIV testing among all women in COMBAT Study (N = 2000)	
No	1135 (56.75)
Yes	865 (43.25)

### Sociodemographic and sexual behaviour factors associated with HIV testing

The socio-demographic characteristics of women experiencing IPV that were significantly associated with HIV testing were age of respondents (χ^2^ = 35.7 p-value<0.001); area of residence (χ^2^ = 23.5, p-value<0.001); years lived in their community (χ^2^ = 10.6, p-value = 0.014); highest level of education (χ^2^ = 42.1, p-value<0.001); ever moved or travelled outside the community for work (χ^2^ = 11.8, p-value = 0.001); and having employment within 12 months preceding the survey (χ^2^ = 6.1, p-value = 0.013). The sexual behaviour factors that showed significant association with HIV testing among respondents from the Pearson’s chi-square test were age at first sex (χ^2^ = 7.5, p-value = 0.024) and ever use of condoms (χ^2^ = 16.5, p-value<0.001). HIV testing was significantly different across various levels of the sexual behaviour characteristics. The socio-demographic and sexual behaviour factors associated with HIV testing among respondents are shown in Table 2.

**Table 2:** Bivariate analysis of factors associated with HIV testing among respondents.

		HIV Testing			
	Total	Not tested	Tested	Chi-square	P-value
**Variables and categories**	**N**	**n (%)**	**n (%)**	** **	** **
No. of respondents	1008	581 (57.64)	427 (42.36)		
**Age (years)**				35.67	<0.001
<25	220	139 (63.18)	81 (36.82)		
25–29	221	101 (45.70)	120 (54.30)		
30–34	182	96 (52.75)	86 (47.25)		
35–39	136	71 (52.21)	65 (47.79)		
40–44	128	85 (66.41)	43 (33.59)		
45–49	121	89 (73.55)	32 (26.45)		
**Area of residence**				23.50	<0.001
Coastal	449	221 (49.22)	228 (50.78)		
Inland	559	360 (64.40)	199 (35.60)		
**Years lived in community**				10.63	0.014
<10	329	173 (52.58)	156 (47.42)		
10–19	221	138 (62.44)	83 (37.56)		
20–29	267	146 (54.68)	121 (45.32)		
>29	191	124 (64.92)	67 (35.08)		
**Highest level of education**				42.05	<0.001
None	227	154 (67.84)	73 (32.16)		
Primary	254	164 (64.57)	90 (35.43)		
Junior high	451	237 (52.55)	214 (47.45)		
Senior high	66	26 (39.39)	40 (60.61)		
Post senior high	10	0 (0.00)	10 (100.00)		
**Current partner’s education level**				3.12	0.210
Same	216	114 (52.78)	102 (47.22)		
Woman more educated	147	83 (56.46)	64 (43.54)		
Male partner more educated	645	384 (59.53)	261 (40.47)		
**Ever moved/ travelled for work**				11.81	<0.001
No	545	341 (62.57)	204 (37.43)		
Yes	463	240 (51.84)	223 (48.16)		
**Employed in the past 12 months**				6.11	0.013
No	171	84 (49.12)	87 (50.88)		
Yes	837	497 (59.38)	340 (40.62)		
**Partner employed**				0.31	0.573
No	974	563 (57.80)	411 (42.20)		
Yes	34	18 (52.94)	16 (47.06)		
**Age at first sex (years)**				7.49	0.024
<16	146	97 (66.44)	49 (33.56)		
16–19	590	341 (57.80)	249 (42.20)		
≥20 / Never had sex	272	143 (52.57)	129 (47.43)		
**First sex partner**				4.97	0.174
Husband	112	54 (48.21)	58 (51.79)		
Boyfriend	777	460 (59.20)	317 (40.80)		
Others	117	67 (56.30)	51 (43.59)		
Never had sex	2	1 (50.00)	1 (50.00)		
**Ever used condom**				16.47	<0.001
No	704	435 (61.79)	269 (38.21)		
Yes	304	146 (48.03)	158 (51.97)		
**No. of main sex partners in last 12 months**				1.43	0.487
0	108	68 (62.96)	40 (37.04)		
1	880	502 (57.05)	378 (42.95)		
>1	20	11 (55.00)	9 (45.00)		
**No. of other sex partners in last 12 months**				3.43	0.179
0	927	535 (57.71)	392 (42.29)		
1	64	33 (51.56)	31 (48.44)		
>1	17	13 (76.47)	4 (23.53)		
**No. of main sex partners in lifetime**				2.55	0.279
0	10	5 (50.00)	5 (50.00)		
1	317	194 (61.20)	123 (38.80)		
>1	681	382 (56.09)	299 (43.91)		
**No. of other sex partners in lifetime**				4.96	0.083
0	813	470 (57.81)	343 (42.19)		
1	127	65 (51.18)	62 (48.82)		
>1	68	46 (67.65)	22 (32.35)		
**No. of transactional sex engaged in**				1.38	0.501
0	753	429 (56.97)	324 (43.03)		
1	75	48 (64.00)	27 (36.00)		
>1	180	104 (57.78)	76 (42.22)		
Self-reported life satisfaction				5.71	0.17
Not satisfied	672	405 (60.27)	267 (39.73)		
Satisfied	336	176 (52.38)	160 (47.62)		
**Any form of IPV in lifetime (n = 2000)**				0.65	0.419
No	992	554 (55.85)	438 (44.15)		
Yes	1008	581 (57.64)	427 (42.36)		

### Predictors of HIV testing among women experiencing IPV

Age, education, employment, residence and condom use and reported satisfaction with life were found to be significant predictors of HIV testing among women experiencing IPV in the study area, after adjusting for confounding factors. Younger women aged 25–39 years were more than twice as likely to test for HIV (AOR:>2.0; 95% CI:1.49–3.52) compared with those above 45 years (Table 3). With regards to education, the likelihood of HIV testing increased with increasing levels of education, although the likelihood of testing among those with primary education was not significantly different from those without formal education. In comparison to respondents without formal education, the odds of HIV testing among women who attained various levels of education were as follows: Primary School (AOR:1.27; 95% CI:0.84–1.93); Junior High School (AOR:2.10; 95% CI:1.42–3.12); Senior High School (AOR:3.87; 95% CI:2.07–7.26); and all 10 respondents with Post Senior High School education (100%) had tested for HIV. Women who were employed during the last 12 months were less likely to test for HIV than those unemployed (AOR:0.66; 95% CI; 0.45–0.96), and those resident in inland areas were also less likely to have an HIV test compared with those living in coastal areas (AOR:1.97; 95% CI: 1.45–2.67). Women who had ever used condoms had a higher odds of testing for HIV than those who had never used condoms (AOR:1.42; 95% CI:1.05–1.93). HIV testing was significantly lower among women whose first sexual encounter was with a boyfriend compared to a husband (AOR: 0.46, 95% CI: 0.29–0.72, p = 0.001). HIV testing was also higher among women who reported satisfaction with their lives (AOR: 1.44, 95% CI: 1.08–1.92, p = 0.014) (Table 3).

**Table 3:** Logistic regression model of predictors of HIV testing among respondents.

Variables and category	COR [95% CI]	P-value	AOR [95% CI]	P-value
**Age group of women**				
<25years	1.00 [reference]		1.00 [reference]	
25–29	2.04 [1.39, 2.98]	<0.001	2.29 [1.49, 3.52]	<0.001
30–34	1.54 [1.03, 2.29]	0.035	2.31 [1.44, 3.68]	<0.001
35–39	1.57 [1.02, 2.42]	0.041	2.41 [1.45, 4.02]	0.001
40–44	0.87 [0.55, 1.37]	0.545	1.46 [0.84, 2.56]	0.183
45–49	0.62 [0.38, 1.01]	0.053	1.10 [0.60, 2.02]	0.765
**Area of residence**				
Coastal	1.87 [1.45, 2.40]	<0.001	1.97 [1.45, 2.67]	<0.001
Rural	1.00 [reference]		1.00 [reference]	
**Years lived in community**				
<10years	1.67 [1.16, 2.41]	0.006	1.60 [1.02, 2.50]	0.039
10–19 years	1.11 [0.74, 1.67]	0.602	1.00 [0.63, 1.60]	0.993
20–29	1.53 [1.05, 2.25]	0.028	1.19 [0.76, 1.89]	0.449
>29	1.00 [reference]		1.00 [reference]	
**Highest level of education**				
None	1.00 [reference]		1.00 [reference]	
Primary	1.16 [0.79, 1.69]	0.449	1.27 [0.84, 1.93]	0.250
Junior high school	1.90 [1.36, 2.66]	<0.001	2.10 [1.42, 3.12]	<0.001
Senior high or above	4.06 [2.34, 7.03]	<0.001	3.87 [2.07, 7.26]	<0.001
**Partner’s education **				
Same	1.32 [0.97, 1.79]	0.082	1.10 [0.78, 1.56]	0.572
Woman more educated	1.13 [0.79, 1.63]	0.495	0.88 [0.59, 1.32]	0.545
Male partner more educated	1.00 [reference]		1.00 [reference]	
**Ever moved/ travelled for work**				
No	1.00 [reference]		1.00 [reference]	
Yes	1.55 [1.21, 2.00]	0.001	1.36 [1.02, 1.80]	0.035
**Employed in the past 12 months**				
No	1.00 [reference]		1.00 [reference]	
Yes	0.66 [0.47, 0.92]	0.014	0.66 [0.45, 0.96]	0.030
**Ever used condom**				
No	1.00 [reference]		1.00 [reference]	
Yes	1.75 [1.33, 2.30]	<0.001	1.42 [1.05, 1.93]	0.024
**First sexual partner**				
Husband	1.00 [reference]		1.00 [reference]	
Boyfriend	0.64 [0.43, 0.95]	0.029	0.46 [0.29, 0.72]	0.001
Others/never had sex	0.72 [0.43, 1.21]	0.219	0.58 [0.32, 1.04]	0.069
**Age at first sex**				
<16years	1.00 [reference]		1.00 [reference]	
16–19	1.45 [0.99, 2.11]	0.058	1.24 [0.81, 1.89]	0.317
20+/Never had sex	1.79 [1.18, 2.71]	0.007	1.13 [0.70, 1.83]	0.606
**Number of main sexual partners in the past 12 months**	1.26 [0.88, 1.81]	0.199	1.21 [0.82, 1.79]	0.334
**Number of times engaged in transactional sex**	0.96 [0.82, 1.13]	0.665	1.13 [0.94, 1.37]	0.193
**Self-reported life satisfaction **				
Not satisfied	1.00 [reference]		1.00 [reference]	
Satisfied	1.38 [1.06, 1.80]	0.017	1.44 [1.08, 1.92]	0.014

Crude and adjusted odds ratios and 95% confidence intervals from logistic regression analyses.

## Discussion

### Prevalence of IPV among women in the study population

Data from the four districts of the Central region of Ghana, which informed the current study revealed that more than half (50.8%) of the women had experienced some form of IPV in their lifetime [16]. This estimate, which compares well with previous estimates of lifetime experience from the GDHS (2008) of 38.7% and the 12-month experience of 27.7% from the GFLHS (2015) [10, 11] suggest continuing perpetuation of IPV against women in Ghana. The reported prevalence of IPV in Ghana, though higher than in other countries in the west Africa sub-region, is within the 15% to 71% range estimated for low- and middle-income countries, obtained from the WHO multi-country study on women’s health and domestic violence [17]. Prevalence of the various types of IPV experienced by study participants include sexual (36.7%), physical (63.8%), economic (20.0%) and emotional (72.8%) with overlapping occurrence. The overlapping occurrence of sexual, physical and other forms of IPV perpetuated against women by their intimate male partners have been well documented globally [6].

### Prevalence of HIV testing among women experiencing IPV

Studies on HIV testing among women experiencing IPV in Ghana are non-existent. However, the prevalence of HIV testing among various population sub-groups in Ghana have been reported to be varied. Tenkorang & Owusu (2010) found a 10% prevalence of HIV testing among women in the general population of Ghana [18]. Secondary analysis of data from the nationally representative, 2014 Ghana Demographic and Health Survey found a low HIV testing prevalence rate of 13% among women and 6% among men aged 15 to 49 years in the 12 months period preceding the survey [19]. Wide variations in HIV testing rates were observed among sub-groups of the population, within regions and across the 10 regions of the country. In that study, the following subgroups had higher HIV testing rates compared with the national average of 13%: Women aged 25–34 years (19%); those married (16%); those with previous STIs (16%), those with only one sexual partner (16%); from richest households (18%); and those with higher than secondary education (26%). Clearly, age, education, marital status, income and sexual behaviour influenced HIV testing among men and women aged 15 to 49 years in the general population of Ghana [19]. The estimates obtained compared well with the previous estimates by Tenkorang & Owusu (2010) [18]. Gyasi & Abass (2018) reported an HIV testing prevalence of 22% among youth in Metropolitan Kumasi of the Ashanti region of Ghana [20]. Although differences in HIV testing rates reported from various studies can be attributed to several factors including study designs, testing settings, study population as well as geographic locations, in general, HIV testing rates in Ghana are considered low, but have been observed to be increasing [21].

The prevalence of HIV testing among women exposed to IPV in the study setting (42.4%) was similar to the prevalence observed among women who had not experienced any form of IPV (44.2%). This was a particularly interesting observation given the documented increased risk of HIV infection among women exposed to IPV. We did not find any documented studies in Ghana or elsewhere reporting similar observations with any plausible explanations. However, potential explanations could include a lack of awareness of the increased risk to HIV infection among women exposed to IPV and/or poor access to HIV testing services, resulting in a low uptake of HIV testing services among these women. Although the observed HIV testing rates among women exposed to HIV in the Central Region are higher than those observed in other areas and sub-groups in Ghana [18–20], the prevalence of HIV testing among women experiencing IPV in the Central region (42.4%) is lower than the reported rate of 82% among women exposed to IPV in Uganda [22]. Uganda has a dual HIV and IPV epidemic with yearly IPV prevalence of 14–33% and lifetime prevalence of 45–54%; and a relatively mature HIV control program that effectively targets at-risk populations and ensures access to HIV testing services through Provider Initiated Testing and Counselling (PITC), Voluntary Counselling and Testing (VCT) and awareness creation through education and information services. Hence, the higher uptake of HIV testing in that population [22]. The relatively low prevalence of HIV testing among women who had experienced IPV in the current study population in Ghana, demonstrates unmet need and gaps in testing services, as a greater proportion of women with IPV, who are at an increased risk of HIV infection, had not had an HIV test at the time of the study. The gaps may exist as a result of inadequate information provided on testing options available to these and other vulnerable groups such as young people and adolescents. It further aligns with the assertion that some women experiencing IPV in their relationships report HIV risk-related behaviours but are often not tested for HIV.

In Ghana, HIV testing services are routinely offered to women attending antenatal care (ANC) clinics through PITC at no cost, towards the prevention of mother-to-child-transmission of HIV, and commercially accessible to the general population on demand, through VCT in public health facilities and private sector pharmacies [19]. Apart from the information and testing services provided to pregnant women during regular ANC services, most vulnerable women, including those exposed to IPV, are not aware of their risk to HIV infection and the need or benefit of testing for HIV. Similarly, knowledge about ART and access to ART among this sub-group of the population and the general population at large, is also poor and hampered by stigma [4, 19]. Thus, it remains essential to provide avenues for women with IPV exposure to obtain HIV test through alternative means such as self-testing and rapid voluntary counselling and testing services within HIV programs. Of critical importance is the need for appropriate health educational intervention messages to increase awareness about the increased risk of HIV infection among women exposed to IPV and the need for regular testing among this vulnerable population. Similarly, knowledge about access to ART and its benefits are essential. The Health Sector must deem it necessary to play a protagonist role in making comprehensive health care available to women imperiled by IPV and serve as an entry point for referring victims for further social support services.

### Predictors of HIV testing among women experiencing IPV

The results of this study indicate that HIV testing among women who experience IPV is influenced by age, education, employment, place of residence, condom use and reported satisfaction with life. Although, there are no comparable studies on HIV testing among women exposed to IPV in Ghana, the findings are consistent with observations from analysis of the nationally representative 2014 GDHS data which identified age, education, marital status, number of sexual partners, household wealth index and region of residence as predictors of HIV testing among women aged 15 to 49 years in the general population of Ghana [19]. Young age, as a predictor of HIV testing, could be related to a lack of knowledge and awareness regarding the vulnerability of IPV exposure to HIV infection as well as poor access to services, which is well documented for young people. Education tends to empower women with literary and analytical skills that enable them to make appropriate health decisions and adopt promotive and protective health behaviours such as condom use and HIV testing. This empirical observation also explains the relationship between condom use and HIV testing. Educated women are also more likely to be economically empowered to access health services. Residence as a predictor of service utilization tends to reflect disparities in access to HIV, IPV and other health and social services in most developing countries, where urban communities tend to have better access to various services, while rural poor communities remain deprived. Other factors found to predict HIV testing among women who experienced IPV in Uganda include knowledge, access to information and decision making to access HIV services [22]. Inadequate testing levels among IPV victims are driven by the assertions that IPV is a risk factor for HIV infection and the fear of violence intimidates women and prevents them from accessing HIV testing services [23]. The relationship between IPV with HIV test result disclosure, for instance, has been shown to be negative as partners who disclosed their HIV test results suffered further and varied forms of IPV [24–26]. Women’s reported satisfaction with life as a predictor of HIV testing might reflect their socioeconomic status and improved accessibility to health services, including HIV test. The high rate of unemployment among partners of women who experienced IPV is noteworthy, given a previous observation from Ghana indicating that women whose husbands were unemployed had 2.41 to 2.58 times the odds of experiencing psychological and physical violence [27]. These observations should strengthen concerns about IPV and its relationship with HIV and the need to address this growing public health problem concurrently.

The findings of this study and others, highlight the importance of sociodemographic characteristics such as women’s education and employment, and behavioural factors such as number of sexual partners, condom use and engagement in transactional sex, among other risky behaviours, which need to be explored to target interventions to improve HIV testing options [28]. To achieve high testing rates in Africa, increasing the availability of testing sites, as well as provider-initiated testing within routine healthcare remain significant interventions. These should be coupled with promoting home-based self-testing to minimize the effect of stigma and related violence, increasing testing centers in communities, as well as decentralized systems for women to know their HIV status, as such systems have been found to be acceptable and accessible to vulnerable groups [29]. These actions are inherent in the efforts to empower women and other vulnerable groups as interventional attempts to reduce the increasing trends of HIV infection and IPV among women [30].

The findings also highlight the need for sensitization campaigns to increase awareness of the importance of HIV testing among women exposed to IPV. This observation should inform programmatic efforts targeted at health education and promotion, women’s empowerment and access to information through media. The diverse interplay of factors influencing HIV testing among women who experience IPV should direct the structuring of interventions to improve access to testing services for this at-risk population and inform a multisectoral approach to reducing the incidence of IPV and improving access to HIV testing among women exposed to IPV.

### Importance of the study and global health implications

The relationship between intimate partner violence and HIV infection is well documented. However, data on the extent of HIV testing among women exposed to IPV and the predictors of HIV testing among these women, which are essential to inform policy and programming to address the dual menace of IPV and HIV is scant, globally, and non-existent in Ghana. This study is the first to document the prevalence and predictors of HIV testing among women exposed to IPV in Ghana and second only to Uganda in the Africa region, where the burden of HIV infection is highest, globally. Both HIV and IPV are global health challenges with gender dimensions. Thus, understanding the factors associated with the uptake of HIV testing services among women exposed to IPV is important. The outcome of this study is expected to influence policy and programming and elicit the design of further studies to unravel the critical issues surrounding the interactions between IPV and HIV testing among women, globally.

### Study limitations

The current analysis used data from the baseline assessment of a cRCT, which assessed the Rural Response System using Community-Based Action Teams in preventing violence against women in four districts of the Central Region of Ghana. Hence, the findings may not be generalizable to women outside the Central Region of Ghana. However, the results offer useful clues about the interactions between IPV and HIV testing among women.

## Conclusions

Despite the documented increased risk of HIV infection among women exposed to IPV [12], this study found no difference in HIV testing rates among women exposed to IPV and those not exposed in the Central Region of Ghana. The observed prevalence of HIV testing among women who experienced IPV in the Central Region of Ghana (42.4%) was considered low in comparison to observations from Uganda, where the lifetime prevalence of HIV testing among women exposed to IPV was 85% [22]. The relatively low uptake of HIV testing among women exposed to IPV in the central region, highlights the need for a holistic and integrated approach to increasing awareness of the interactions between HIV and IPV, and to improve access to HIV testing among women experiencing IPV, which remain critical to HIV control efforts. The identified predictors of HIV testing among IPV exposed women, reflect the effects of gender imbalance and attendant socioeconomic vulnerabilities, which impact the ability of women to access health and other social services and make informed decisions regarding their health. These observations point to a need for expanding access to HIV and IPV related services, using multi-sectoral approaches to mitigate the socioeconomic and behavioural factors, which hamper HIV testing among women experiencing IPV. Integration of HIV testing and IPV support services is expected to augment the combination prevention strategies that curb the incidence of IPV and improve the utilization of HIV testing services. This study provides a compelling evidence base for informing policy and programming at various levels to improve HIV testing among women experiencing IPV. Gender-based interventions targeting specific IPV forms and considering contextual factors should be employed in addressing the dual problem of HIV infection and IPV among women as urgent intertwined public health problems.
